# Whey Proteins–Zinc Oxide Bionanocomposite as Antibacterial Films

**DOI:** 10.3390/pharmaceutics13091426

**Published:** 2021-09-08

**Authors:** Paolo Pino, Silvia Ronchetti, Chiara Mollea, Marco Sangermano, Barbara Onida, Francesca Bosco

**Affiliations:** Department of Applied Science and Technology, Politecnico di Torino, 10129 Turin, Italy; paolo.pino@polito.it (P.P.); silvia.ronchetti@polito.it (S.R.); chiara.mollea@polito.it (C.M.); marco.sangermano@polito.it (M.S.); francesca.bosco@polito.it (F.B.)

**Keywords:** nanocomposite, antibacterial, wound dressing

## Abstract

The use of toxic crosslinking agents and reagents in the fabrication of hydrogels is a frequent issue which is particularly concerning for biomedical or food packaging applications. In this study, novel antibacterial bionanocomposite films were obtained through a simple solvent casting technique without using any crosslinking substance. Films were made from a flexible and transparent whey protein matrix containing zinc oxide nanoparticles synthesised via a wet chemical precipitation route. The physicochemical and functional properties of the ZnO nanoparticles and of the composite films were characterised, and their antibacterial activity was tested against *S. epidermidis* and *E. coli*. The synthesised ZnO nanoparticles had an average size of about 30 nm and a specific surface area of 49.5 m^2^/g. The swelling ratio of the bionanocomposite films increased at basic pH, which is an appealing feature in relation to the absorption of chronic wound exudate. A n-ZnO concentration-dependent antibacterial effect was observed for composite films. In particular, marked antibacterial activity was observed against *S. epidermidis.* Overall, these findings suggest that this novel material can be a promising and sustainable alternative in the design of advanced solutions for wound dressing or food packaging.

## 1. Introduction

The preparation of in-situ forming hydrogels often requires the use of toxic cross-linking agents or precursor monomers [1,2,3]. In other cases, toxic substances are generated from materials degradation [4]. For applications such as wound dressing or food packaging, this is not acceptable, and novel non-toxic and biodegradable materials and processes are needed, as well as materials with smart and antimicrobial properties that can prevent wound infection or food spoiling [5,6,7,8]. This function is often achieved by combining hydrogel-based materials with antimicrobial agents, such as antibiotics. Most recently, hydrogels have been used in combination with nanostructured materials to produce innovative nanocomposites exploiting the promising antimicrobial properties of some materials, including metals, oxides, and carbon-based materials, at the nano-scale [9,10,11]. However, the synthesis of these nanostructured antimicrobials often requires the use of toxic solvents and reagents as well.

Whey proteins, particularly in the form of Whey Protein Concentrate (WPC) and Whey Protein Isolate (WPI), are biocompatible and biodegradable biomolecules abundantly available as by-products of dairy production. Due to their nutritional properties, they have been largely researched for food and biomedical applications, such as drug encapsulation, controlled release and food packaging [12,13,14]. 

Whey proteins show excellent gelation capabilities that do not involve the use of toxic chemicals and possess good mechanical, water absorption and gas barrier properties as well [15,16,17]. These characteristics make whey proteins excellent candidates for the development of new hydrogel systems. Recently, α-lactalbumin—the second major constituent of whey proteins after β-lactoglobulin—was electrospun by Guo and co-workers into nanofiber dressings for wound healing [18].

Nanostructured zinc oxide (ZnO) is an interesting and well-known nanomaterial with numerous applications in the biomedical field [19,20,21,22]. It is also an effective antimicrobial agent [23,24], acting through three main bactericidal mechanisms: release of Zn^2+^ ions, UV-enhanced production of Reactive Oxygen Species (ROS) and physical interactions with microbial cells, which are in turn influenced by the nanoparticle morphology and size [25,26,27,28]. It is frequently investigated for fabrication of nanocomposites for food packaging [29], antimicrobial coatings [30] and wound dressings [31,32,33].

In this study, the design and characterisation of a novel bionanocomposite film based on whey proteins and nano-sized ZnO is described, and its antibacterial properties against *Escherichia coli* and *Staphylococcus epidermidis* were tested for the first time. A simple wet chemical precipitation route was used for ZnO synthesis, leading to spherical nanoparticles. Whey protein films were obtained through a cheap solvent casting technique, allowing for the incorporation of variable zinc oxide content. The antibacterial properties of zinc oxide were also assessed independently from the film. Characterization of the films allowed the gaining of insights into the material, its applications and the interactions between the two phases.

## 2. Materials and Methods

### 2.1. Materials

Zinc acetate di-hydrate (≥98%), potassium hydroxide (≥85%), absolute ethanol and glycerol (≥99%) were purchased from Sigma-Aldrich (St. Louis, MO, USA) and used as received. Whey Protein Isolate (WPI) and Whey Protein Concentrate (WPC) powders were kindly supplied by Milei GmbH (Leutkirch im Allgäu, Germany). Mueller Hinton Broth (MHB) and Mueller Hinton Agar (MHA) were supplied by OXOID Ltd (Basingstoke, United Kingdom). Phosphate Buffered Saline (PBS) tablets were purchased from Sigma-Aldrich (St. Louis, MO, USA).

### 2.2. Synthesis of Nanostructured ZnO

Nanostructured zinc oxide (n-ZnO) was obtained through a slightly modified wet chemical precipitation method previously described [34], based on the work of Mitra and co-workers [35]. Briefly, 14.75 g of zinc acetate dihydrate and 7.4 g of potassium hydroxide were dissolved in 60 mL and 32 mL of ethanol respectively. The two solutions were then mixed and kept at 60 °C for 72 h under constant stirring and reflux. This induced the precipitation of a white powder (about 4 g) that was recovered by means of a 30 min centrifugation cycle at 4000 rpm and subsequently washed three times with ethanol. 

### 2.3. Characterization of ZnO Particles

The obtained n-ZnO was characterised through X-ray diffraction (XRD) using a PANalytical X’Pert Diffractometer (Cu Kα radiation, Almelo, The Netherlands). Field Emission Scanning Electron Microscopy (FESEM) was carried out with a ZEISS Merlin instrument (Oxford Instruments, Abingdon-on-Thames, UK). ImageJ software (open source, https://imagej.net/, accessed 26 August 2021) was used to measure particle size distribution by analyzing 62 particles. Nitrogen physisorption measurements were performed using a ASAP2020 Plus Micromeritics apparatus (Norcross, GA, USA). Prior to the measurements, samples were degassed at 70 °C for 2 h. Specific Surface Area (SSA) was determined based on the Brunauer–Emmet–Teller (BET) model in the relative pressure p/p^0^ range of 0.01-0.1. Zeta potential and particle size distribution were obtained through Dynamic Light Scattering by means of a Malvern Instruments DLS Zetasizer Nanoseries ZS90 instrument (Malvern, UK). For these measurements, aqueous 0.5 mg/mL n-ZnO suspensions were prepared at pH of 3, 5 and 7 and sonicated in an Elmasonic ultrasonic bath (Elma Schmidbauer GmbH, Singen, Germany) for 1 h before testing. All measurements were repeated three times.

### 2.4. Preparation of Whey Protein Films

Protein-based films were fabricated by means of a simple solvent casting technique. An aqueous WPI or WPC film forming suspension (FFS) was prepared with a 10.4% (*w/w*) protein concentration by dispersing WP powder in distilled water at room temperature under constant agitation. The pH of the suspension was adjusted to 7 with the addition of 1 M NaOH solution. The suspension was then placed inside a Huber 112A-E thermostatic bath (Huber, Offenburg, Germany) for 20 min at 70 °C under continuous magnetic stirring to induce protein denaturation. Subsequently, the FFS was cooled down to ambient temperature and 60% (*w/w*) of glycerol was added on a dry basis as a plasticizing agent. A volume of suspension was then casted into circular non-stick moulds such that the volume-to-surface ratio was kept equal to 0.135 m, and finally dried in an ISCO 900 oven for 2.5 h at 60 °C. This procedure was refined after multiple iterations conducted at different temperatures, pH values and treatment times to explore the effect of process parameters on gelling phenomena. Thickness of the films was measured with a thickness gauge. 

### 2.5. Preparation of WP-ZnO Films

For the preparation of composite films, aqueous suspensions of n-ZnO with concentrations of 20, 40 and 60 mg/mL were prepared and sonicated in an Elmasonic ultrasonic bath (Elma Schmidbauer GmbH, Singen, Germany) for 60 min and subsequently vigorously mixed with a magnetic stirrer with the whey protein FFS obtained as illustrated above, prior to casting.

Films with n-ZnO percentage mass fraction of 2%, 4% and 6% with respect to the dry weight of WPI (or WPC) in the FFS were thus obtained and labelled WPI–ZnO-2, WPI–ZnO-4 and WPI–ZnO-6 respectively. Two additional films with 7.5% and 15% *w/w* n-ZnO content were also prepared for antibacterial testing.

### 2.6. Characterization of the Films

Composite and non-composite films were characterised by means of X-Ray Diffraction using a PANalytical X’Pert Diffractometer (Cu Kα radiation, Almelo, The Netherlands). Spectroscopic characterization was carried out by means of UV–Vis spectroscopy, using a Lambda 465 UV/Vis spectrophotometer (PerkinElmer, Waltham, MA, US), as well as Attenuated Total Reflectance Fourier-Transform Infrared Spectroscopy (ATR-FTIR), using a Nicolet iS50 FTIR Spectrometer equipped with Smart iTX optics and diamond crystal (Thermo Fischer Scientific, Waltham, MA, USA). Mechanical properties were assessed using an Instron 3366 Universal Testing System (Norwood, MA, USA). WPI, WPI–ZnO-2, WPI–ZnO-4 and WPI–ZnO-6 were cut into rectangular 3 × 10 mm specimens. Grip separation speed was set to 2 mm/min.

### 2.7. Swelling Tests

Swelling tests were carried to assess the ability of the material to absorb water and PBS (pH = 7.4). All films were cut into 1.8 cm diameter disks and weighed. Disks were then dried at 60 °C inside an oven until stabilization of mass at a constant value. Films were then immersed in 20 mL of PBS or distilled water at different pH levels, namely 5, 7, 9 and 11. Water pH was titrated with NaOH and HCl solutions. After 30 min intervals, samples were collected, blotted from excess water and weighed. The procedure was repeated until stabilization of the measured mass. The swelling ratio (SR) of the samples was determined as:

(1) $$\mathrm{SR}=\frac{m_{w}-m_{d}}{m_{d}}\cdot 100$$

where m_w_ is the wet mass of each sample and m_d_ is the dry mass. Each experiment was repeated in triplicate.

### 2.8. Antibacterial Activity

The antibacterial activity of n-ZnO powders and bionanocomposite films was assessed against Gram-positive *Staphylococcus epidermidis*, LMG 10474 and Gram-negative *Escherichia coli,* LMG 08063 bacteria. Mueller Hinton Broth (MHB) and Mueller Hinton Agar (MHA) were used as test media in accordance with standard procedures, as described below.

### 2.9. Antibacterial Activity of n-ZnO

The antibacterial activity of n-ZnO was tested by means of a modified Kirby–Bauer agar diffusion test following the standard “EUCAST disk diffusion method” (2021) [36] with some adaptations. 

A standardised inoculum, corresponding to 10^6^ CFU/mL, was obtained by dispersing three microbial colonies from an overnight culture at 37 °C on MHA into 5 mL of sterile 0.85% *w/v* NaCl solution (10^8^ CFU/mL) and diluting (1:100) the suspension with MHB.

ZnO samples were prepared, in sterile conditions, by dispersing the n-ZnO in distilled water (pH = 6) inside microcentrifuge tubes (capacity 2 mL). The following n-ZnO concentrations were tested: 25, 20, 17.5, 15, 10, 7.5, 6, 5, 3.75, 1.92 and 0.96 mg/mL. All the suspensions were sonicated for 60 min at 35 kHz to break down agglomerates and to further disperse n-ZnO particles.

A standardised inoculum, 100 μL, was spread over the surface of MHA Petri dishes. Subsequently, 20 μL of each n-ZnO suspension was deposited as single droplets equally spaced around the circumference of the Petri dish, while a 20 μL droplet of pure water was deposited in the centre of the dish as a control.

Petri dishes were incubated at 37 °C for 24 h. All experiments were performed in triplicate. At the end of the incubation, samples were observed and inhibition halos were measured as the average distance between the drop outline and that of the bacterial growth, at the two perpendicular diameters (mm).

### 2.10. Antibacterial Activity of Bionanocomposites Films

A disk diffusion method was adopted to assess the antibacterial activity of bionanocomposite films; this test was also set up by modifying the standard “EUCAST disk diffusion method” (2021) [36]. A WP film without n-ZnO was used as positive control. Both the top and the bottom face of each film were tested against *S. epidermidis* and *E. coli* to assess the influence of potential different n-ZnO surface concentrations due to the film preparation process on antibacterial activity. Films were cut into 18 mm diameter disks and gently placed onto the surface of MHA Petri dishes, previously inoculated with 100 μL of a standardised bacterial inoculum prepared as described in Section 2.9. Samples were subsequently incubated at 37 °C for 24 h. All experiments were performed in triplicate. At the end of the incubation period, the inhibition zone was measured as the average distance between the edge of the disk and that of the bacterial growth, at the two perpendicular diameters.

## 3. Results and Discussion

### 3.1. Characterization of n-ZnO

**Figure 1:**
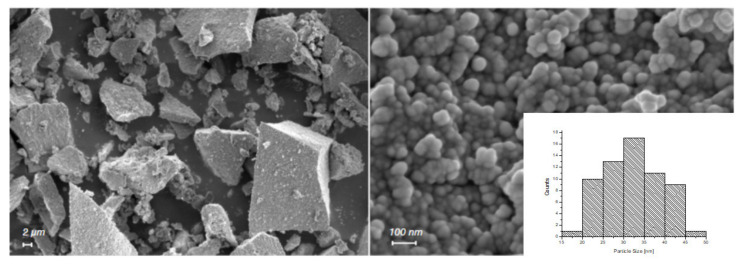
FESEM image of n-ZnO at 5K (**left**) and 250K (**right**) magnification. Particle size distribution is shown in the bottom right corner.

FESEM images reported in Figure 1 show the n-ZnO sample, which appears in the form of micrometric irregular aggregates constituted of spherical primary nanoparticles with an average diameter of approximately 30 nm. The calculated particle size distribution (inset in Figure 1, bottom-right side) revealed an average diameter of 32 nm.

**Figure 2:**
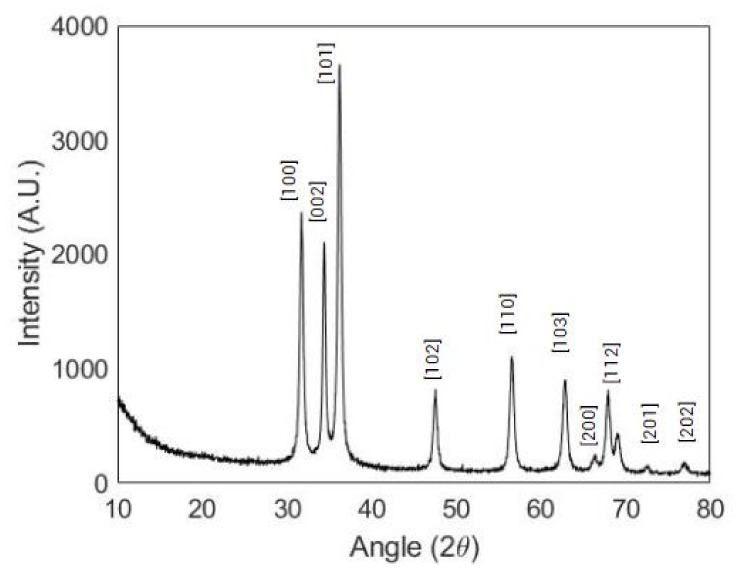
XRD pattern of n-ZnO.

The XRD spectrum is reported in Figure 2 and reveals that n-ZnO was characterised by a hexagonal, wurtzite-type crystalline structure (JCPDS ICDD 36-1451).

**Figure 3:**
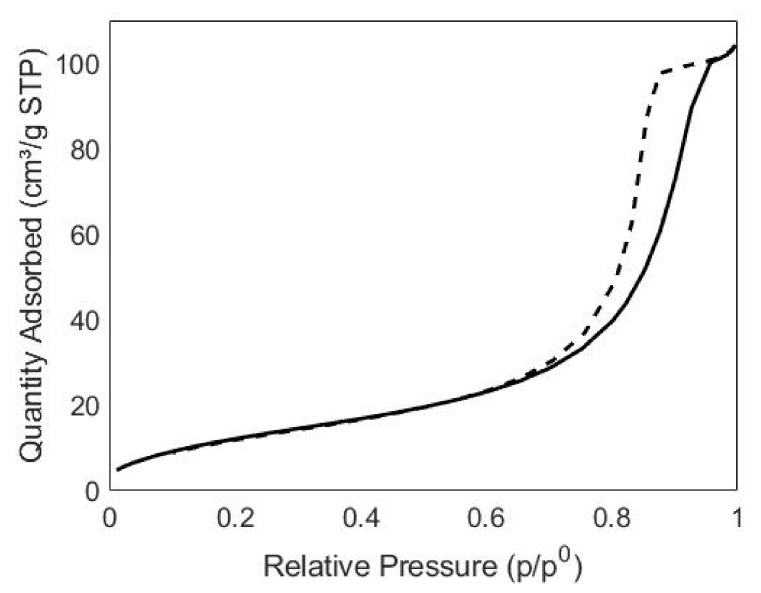
Nitrogen sorption isotherms (adsorption branch— desorption branch ---) of n-ZnO.

Nitrogen physisorption measurements (Figure 3) led to the determination of a SSA equal to 49.5 m^2^/g. The isotherm was type IV (IUPAC classification), revealing the presence of mesopores, most likely due to the interstices between the particles, as confirmed by FESEM images.

These measurements confirm that the n-ZnO sample was similar to the one previously obtained [34] using methanol as solvent (SSA = 75 m^2^/g, particle size in the range 30–60 nm, hexagonal wurtzite-type phase), thereby showing that the same synthesis can be reproduced successfully in a greener and safer way by replacing methanol with ethanol.

**Figure 4:**
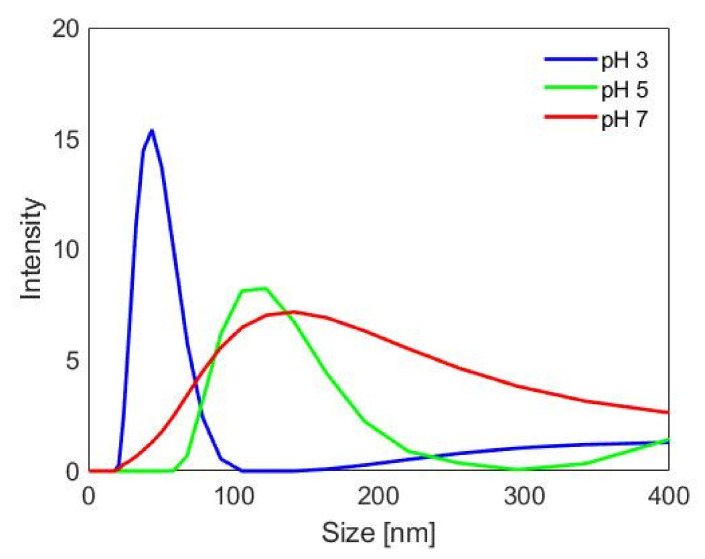
n-ZnO particle size distribution curves in aqueous suspensions at different pH values.

The size distribution curves obtained by means of DLS are illustrated in Figure 4. They show the occurrence of aggregation of n-ZnO depending on the pH of the aqueous nanoparticles suspension. As can be seen in the figure, pH 3 was the condition under which n-ZnO were less aggregated, as revealed by the sharp and intense peak registered at 48 nm. This is in good agreement with the nanoparticle size provided by FESEM imaging if the hydrodynamic radius is taken into account. At higher pH values, peaks appeared increasingly lower and wider, reaching a maximum width at pH 7. This is explained by the results obtained by Berg and co-workers [37] locating the isoelectric point of 30 nm ZnO nanoparticles at pH of 7. In fact, at the isoelectric point, the lack of electrostatic repulsion may favour nanoparticle aggregation. This pH value coincides with that of the whey protein FFS to which n-ZnO was added upon preparation of the nanocomposite films, as described above. This analysis therefore suggests that particular attention should be devoted to the preparation of the nanoparticle suspension at this stage in order to prevent excessive agglomeration from occurring.

### 3.2. Characterization of the Films

**Figure 5:**
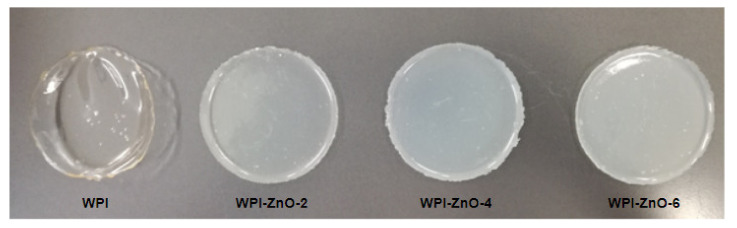
WPI films with increasing n-ZnO concentrations.

Figure 5 reports pictures of composite and non-composite WPI films. WPI films possessed higher transparency with respect to their WPC equivalents (Figure S1), which instead appeared yellowish due to the higher content of impurities in the concentrate mixture, such as fats and phospholipids [15]. These molecules were also considered responsible for lower mechanical properties in WPC films, as these largely depend on β-lactoglobulin content [38,39]. For this reasons WPI was the mixture of choice for the further investigation of antimicrobial composites. The addition of zinc oxide caused opacity and a white coloration of the films, a common feature of biopolymer–ZnO composites [9,40,41]. Average film thickness was approximately 60 μm. 

Mechanical properties of the films were also investigated and tensile strength, Young’s modulus and elongation at break values are reported in Table 1. The addition of n-ZnO produced a marked increase in the elastic modulus, which increased with higher filler content. This effect is commonly reported as a consequence of the physical interaction between the two phases [32,42]. Tensile strength reached a maximum for n-ZnO concentration of 2% *w/w*. Elongation at break fell dramatically after zinc oxide addition, and decreased with increasing nanoparticles content. This can be ascribed to the increased amount of discontinuities and aggregates in the material, which promote nucleation and propagation of cracks by creating localised stress concentrations [43,44].

It is here worth noting that the elastic modulus and elongation at break values measured fall within the range of human skin [45]. Elastic modulus has been also inversely correlated to the swelling ability of biopolymers [46].

**Figure 6:**
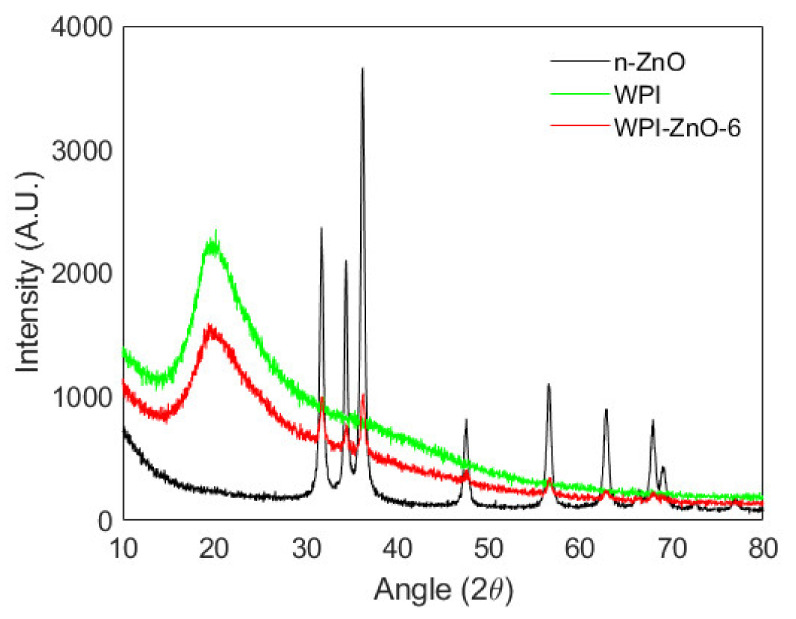
XRD spectra of n-ZnO (black), WPI (green) and WPI–ZnO-6 composite film (red).

Figure 6 reports the XRD pattern of the WPI film, which was typical of amorphous materials. The composite WPI–ZnO-6 film pattern showed distinctive features of both continuous and dispersed phases, with characteristic peaks of ZnO overlapped to the spectrum of the amorphous matrix. No alterations to either the WPI or the ZnO patterns could be appreciated, suggesting that nanoparticles do not favour ordering or crystallization of the protein matrix upon mixing with the film-forming suspension. Similar results were obtained for the nanocomposite films with lower n-ZnO content (reported in Figure S2).

**Figure 7:**
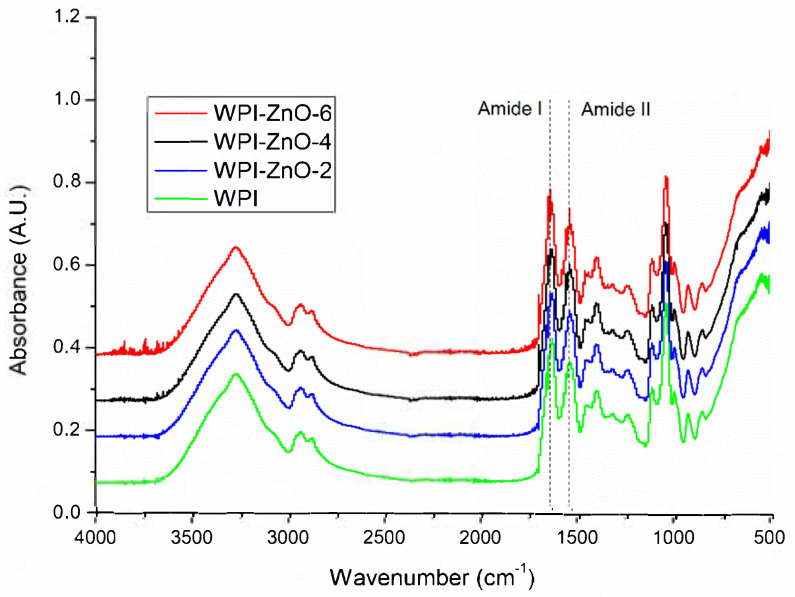
ATR-FTIR Spectra of WPI and WPI–ZnO films.

In the ATR-FTIR spectra shown in Figure 7, the most specific absorbance bands for proteins can be identified, i.e., those related to the Amide I (υ C=O; υ C–N) and Amide II (δ N–H; υ C–N) bonds at 1640 cm^−1^ and 1540 cm^−1^, respectively. Other peaks were present in the region between 1500 cm^−1^ and 700 cm^−1^ that can be ascribed to fatty acids and carbohydrates contained in WPI [47]. No shift in the bands was observed upon increasing n-ZnO content in the composite film, possible evidence of interactions between the matrix and the filler [48,49].

### 3.3. Swelling Tests

**Figure 8:**
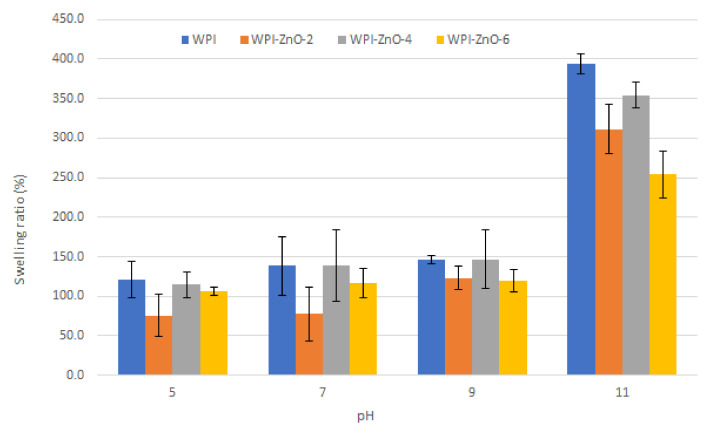
Swelling Ratio versus pH of the WPI film and WPI–ZnO films with different ZnO contents.

Figure 8 reports the results of swelling tests, i.e., the values of the swelling ratio obtained at different pH of the immersion solution. For both WPI and WPI–ZnO films the swelling ratio increased slowly as pH increased from 5 to 9 and then increased rapidly at pH higher than 9.

The analogous behaviour observed for WPI and WPI–ZnO films suggested that the swelling process was mostly dictated by the protein matrix. At pH values close to the isoelectric point of whey proteins (~5) [14], the effect of reciprocal electrostatic repulsion forces was absent and interaction is dominated by hydrogen bonds. This promoted the contraction of the matrix and prevented water molecules from being absorbed, thereby determining the smallest SR.

At high pH (above 9), full deprotonation of carboxylic moieties occurred, thus maximizing electrostatic repulsion and favouring water uptake [50,51]. This appeared to be most evident for the WPI film. 

It is worthwhile to mention that pH also plays a role in whey protein denaturation and gelation by governing protein structures unfolding, as previously reported [52,53]. This could therefore apply to the residual non-denatured and non-gelled protein fraction of the films, ultimately affecting their ability to swell. 

The effect of n-ZnO was an overall decrease in SR, which could mostly be appreciated at high pH values (>9), where the presence of nanoparticles yielded a smaller SR increase. This can be attributed to physical interactions between the matrix and the n-ZnO, with the nanoparticles increasing the Elastic modulus and hindering the expansion of the protein matrix by limiting the required structural rearrangement of the biomacromolecules [31,46,54].

It is also worth highlighting that the WPI–ZnO-4 sample showed a higher SR compared to its WPI–ZnO-2 and WPI–ZnO-6 counterparts. This may be explained by considering the effect of the osmotic pressure. A small nZnO content (2%) may not be sufficient to generate enough osmotic pressure to promote water uptake by the hydrogel [54], which instead may occur at higher nanoparticle content (4%). When additional ZnO is introduced (6%), however, the stronger physical hindrance to expansion and the occupation of matrix free space by the more abundant nanoparticles became the dominating phenomena affecting swelling. Considering the observed SR values, we can compare the one at neutral pH with data reported in the literature. The value of the WPI film (~140%) was lower than those reported by Wahid and co-workers [55] for a carboxymethyl–chitosan hydrogel (~600%) and by Namazi [54] for an oxidised-starch (~2400%) matrix, while it was higher than that reported for β-chitin (~22%) by Kumar [56] and for keratin–chitosan (~23%) by Zhai [57]. For the keratin–chitosan hydrogel, addition of ZnO caused a reduction in the SR at the same neutral pH, similarly to what was herein observed for WPI–ZnO film. ZnO instead induced an SR increase in the carboxymethyl–chitosan hydrogel and in the oxidised-starch one, thanks to the buildup of osmotic pressure inside the matrix. However, in these cases, SR also decreased above a ZnO content threshold, due to the abovementioned hindrance to the swelling. In these two latter cases, moreover, SR decreased with increasing pH value, conversely to the WPI case.

The low variability of SR with n-ZnO content at pH values close to neutrality in the WPI–ZnO composite can allow tuning of the amount of nanoparticles without significantly affecting the swelling function of the material, thus optimizing mechanical, optical and gas barrier properties, which are particularly relevant for food packaging applications. Meanwhile, the marked increase in SR at pH > 9 can be exploited as a sharp stimuli-responsive function and enable novel applications.

**Figure 9:**
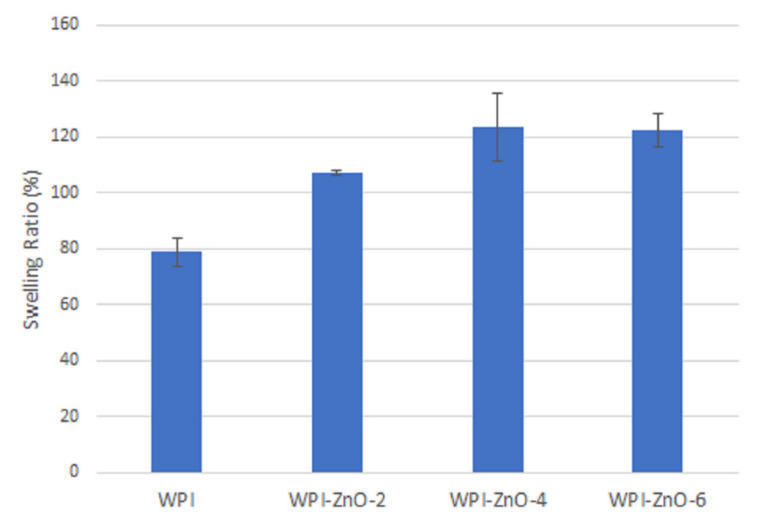
Swelling ratio of WPI and WPI–ZnO composites in PBS.

Concerning potential applications in wound healing, the increased swelling ability at high pH values is an appealing feature for the absorption of chronic wound exudate, which typically ranges from pH 7.5 to 8.9 [58]. In this context, swelling ratios of WPI and WPI–ZnO films were additionally measured in PBS and results are reported in Figure 9. The value for WPI film (79 ± 5%) was lower than the corresponding value in water at neutral pH (Figure 8). This can be ascribed to the higher ionic strength of the PBS solution, which reduced the range of repulsive electrostatic interactions within the matrix network [4,54]. The addition of n-ZnO was instead found to cause an increase in the swelling ratio [49], presumably due to the abovementioned buildup of osmotic pressure. Within the context of wound healing applications, this is a positive feature, in that a higher content of n-ZnO can therefore lead to both higher wound exudate absorption as well as more significant antibacterial effects, as will be shown in the following sections.

### 3.4. Antibacterial Activity of n-ZnO

**Figure 10:**
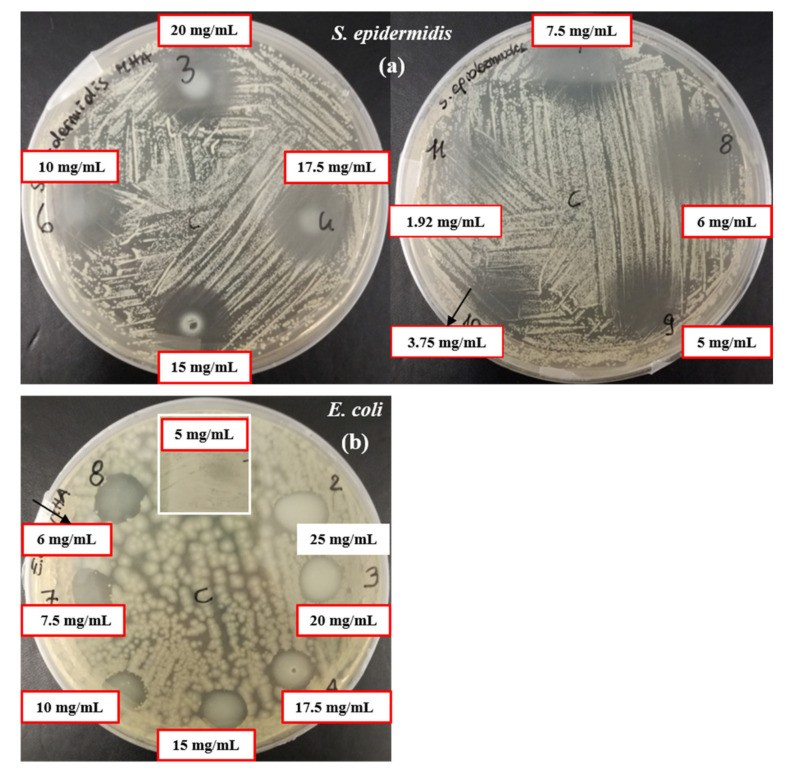
Modified Kirby–Bauer agar diffusion test against *S. epidermidis* (**a**) and *E. coli* (**b**). Red frames indicate the n-ZnO concentrations tested against both microorganisms. Black arrows indicate the lowest concentrations of ZnO that inhibit the bacterial growth, 3.75 and 6 mg/mL, for *S. epidermidis* and *E. coli* respectively.

The antibacterial activity of n-ZnO against *S. epidermidis* and *E. coli* was preliminarily assessed by means of a modified Kirby–Bauer agar diffusion test and the results are shown in Figure 10.

As far as *S. epidermidis* is concerned, at the lowest tested concentration, 1.92 mg/mL, a growth reduction was observed, while at concentrations from 3.75 mg/mL to 20 mg/mL total growth inhibition was obtained. By comparison, for *E. coli*, a n-ZnO concentration of 6 mg/mL was necessary to inhibit bacterial growth. Considering the lower sensitivity of *E. coli*, concentrations below 5 mg/mL were not tested, while the concentration of 25 mg/mL was evaluated as the highest one.

**Figure 11:**
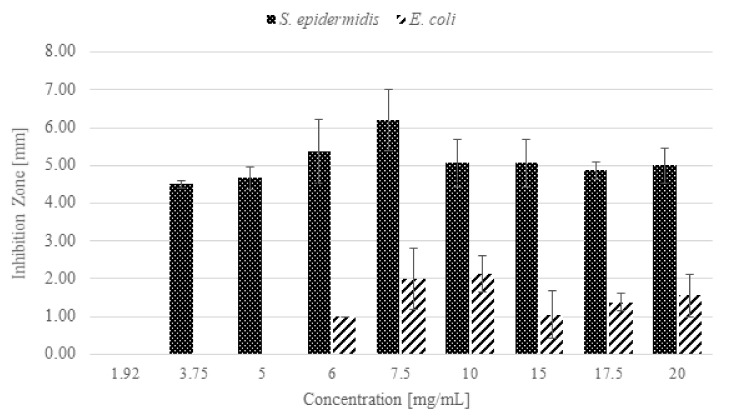
Inhibition zones from the modified Kirby-Bauer agar diffusion test against *S. epidermidis* and *E. coli*.

From the inhibition halo values, reported in Figure 11, it is possible to observe that, under the tested conditions, n-ZnO had a higher antibacterial activity against *S. epidermidis* than against *E. coli*. The result is evident, for example, at the n-ZnO concentration of 6 mg/mL: the inhibition halo measured for *S. epidermidis* corresponded to 5.38 mm, while that of *E. coli* was less than one-fifth the size (approximately 1 mm). The maximum inhibition halo measured for *S. epidermidis* was 6.21 mm, whereas for *E. coli* it was only 2.13 mm. This result is consistent with the literature [22,59,60,61,62] reporting a higher activity of ZnO on Gram-positive bacteria. This is generally attributed to the absence in Gram-positive species of the outer membrane typical of Gram-negative species [63]. It should be mentioned, however, that in some cases ZnO nanoparticles have shown higher efficacy against Gram-negative species [64,65].

### 3.5. Antibacterial Activity of Bionanocomposite Films

Once the antibacterial activity of the n-ZnO was ascertained, disk diffusion tests were performed to assess the antibacterial efficacy of WPI films containing n-ZnO, and WPI films prepared without n-ZnO were used as controls.

Table 2 shows the inhibition halo values measured for WPI–ZnO-2, WPI–ZnO-4 and WPI–ZnO-6 against *S. epidermidis*. A concentration dependent antibacterial activity can be observed. Although the highest inhibition was obtained with the WPI–ZnO-6 film, the significant overlap—within uncertainty—between WPI–ZnO-4 and WPI–ZnO-6 samples suggested that WPI–ZnO-4 could be an optimal compromise between zinc oxide content and antibacterial efficiency. Very high n-ZnO contents can in fact lead to reduced swelling and nanoparticle agglomeration, in turn worsening mechanical properties. An optimised ZnO nanoparticle content can also help avoid potential cytotoxic effects on skin cells, which are still debated in the literature [66,67].

As a general consideration, the antimicrobial effect of the films was attributed to the presence of n-ZnO, as no inhibition occurred in the WPI control film. Inhibition halos against *S. epidermidis* were comparable with those reported in other studies by Azari [68], Namazi [54] and Arfat [69] using ZnO nanoparticles of similar sizes, shapes and concentrations combined with beta-glucan, oxidised starch, and fish protein isolate/fish skin gelatin films respectively, and testing the nanocomposites against other Gram-positive species. In the present study, *S. epidermidis* was chosen due to being a very common species in the human skin microbiota, and responsible for numerous skin wounds, especially nosocomial and surgical ones [70]. Moreover, this strain has been inadequately investigated in the literature for similar applications with ZnO-containing bionanocomposite materials. The antibacterial activity obtained against the Gram-positive bacteria was also comparable with that of other WPI-based nanocomposites. Sani et al. [71,72] developed WPI films embedded with TiO_2_ nanoparticles (1% *w/w*) and cellulose nanofibers, obtaining inhibition halos of 4.00 ± 0.55 and 4.10 ± 0.68 mm against *L. monocytogenes* and *S. aureus* respectively. Karimi and co-workers [73] obtained inhibition halos of 0 and 3.5 ± 1.0 mm against the same species for WPI/polydextrose films incorporated with cellulose nanofibers and *L. plantarum,* a Gram-positive bacteria here employed for its antagonistic effect against the growth of other pathogens. Mohammadi [13] obtained an inhibition halo of 2.00 ± 0.61 mm against *S. aureus* with a WPI film containing chitosan nanofibers and nanostructured lipid carriers loaded with cinnamon essential oil. In the present study, no intrinsic antibacterial activity of WPI was reported, similarly to the abovementioned works.

**Figure 12:**
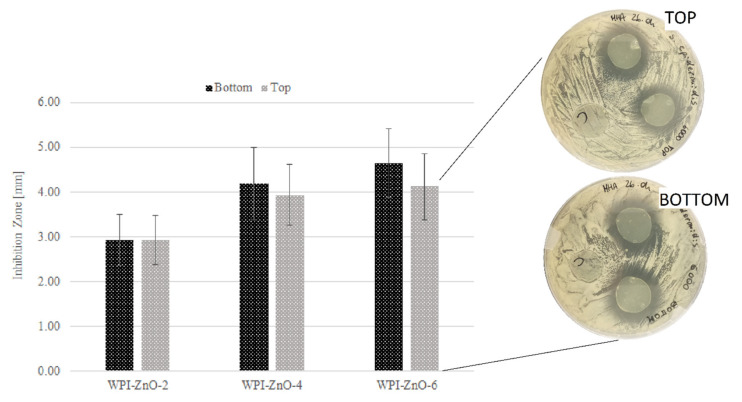
Effect on the inhibitory activity of WPI–ZnO films of top and bottom faces against *S. epidermidis*.

In Figure 12, results obtained by testing the top and the bottom film faces against *S. epidermidis* are shown. For all samples, it was not possible to identify a significant difference between the two film faces. This suggests that n-ZnO maintained good dispersion across the thickness of the film. This could translate into orientation-independent, stable and sustained antimicrobial activity even during film degradation, thus ensuring optimal levels of efficacy throughout the whole life of the device in applications such as wound healing or food packaging.

Concerning antibacterial activity against *E. coli*, no inhibition was observed for WPI–ZnO-2, WPI–ZnO-4 and WPI–ZnO-6. For this reason, WPI–ZnO films with 10% and 15% *w/w* oxide contents were prepared and tested (results not shown). In these two cases, 0.65 ± 0.61 and 1.25 ± 0.60 mm inhibition halos were measured respectively when the bottom faces of the film were tested. Top faces showed comparable results.

In contrast with these results, Hezma [43] and Thanusha [74] obtained a greater effect on Gram-negatives than on Gram-positives using chitosan/poly(vinylalcohol) and gelatin/hyaluronic acid/chitosan matrixes respectively. The previously cited studies on WPI-based nanocomposites also reported higher effects against *E. coli* [13,71,72,73].

## 4. Conclusions

This paper described a simple procedure to produce antibacterial bionanocomposite films using whey proteins and nanostructured zinc oxide. Zinc oxide was obtained from a modified wet chemical precipitation route, showing that methanol could effectively be replaced by ethanol without significantly altering the product of the synthesis and the physicochemical properties of the material. This, coupled with the environment-friendly nature of whey proteins, can ultimately lead to green and sustainable chemical processes and materials. The zinc oxide nanoparticles also showed good antibacterial activity, especially against *S. epidermidis*. Characterisation analyses revealed a successful inclusion of n-ZnO within the protein matrix. ATR-FTIR, XDR and swelling tests suggested that only weak physical interactions existed between the two phases of the composite.

A marked antibacterial effect was observed for the composite films against *S. epidermidis* with small amounts of n-ZnO, whereas higher zinc oxide concentrations were required to generate a weak response on *E. coli*. As a whole, the results showed that whey proteins–zinc oxide bionanocomposites are promising candidates as antibacterial films to be applied in wound healing and food packaging.

## Figures and Tables

**Table 1:** Mechanical properties of WPI and WPI–ZnO films.

Sample	Young’s Modulus [MPa]	Tensile Strenght [MPa]	Elongation at Break [%]
WPI	12.65 ± 0.99	0.56 ± 0.18	106.16 ± 15.97
WPI–ZnO-2	32.27 ± 1.37	0.71 ± 0.15	44.19 ± 4.71
WPI–ZnO-4	36.25 ± 1.47	0.69 ± 0.04	30.90 ± 2.73
WPI–ZnO-6	39.34 ± 3.30	0.65 ± 0.10	14.67 ± 2.95

**Table 2:** Inhibition halos of bionanocomposites against *S.epidermidis*.

	WPI	WPI–ZnO-2	WPI–ZnO-4	WPI–ZnO-6
Inhibition halo [mm]	0	2.94 ± 0.57	4.19 ± 0.81	4.66 ± 0.77

## Supplementary Materials

The following are available online at https://www.mdpi.com/article/10.3390/pharmaceutics13091426/s1, Figure S1: UV-vis spectra of WPI and WPC films. Figure S2: XRD spectra of ZnO and of WPI and WPI–ZnO films.

## Abbreviations

ZnO: Zinc Oxide; ROS, Reactive Oxygen Species; n-ZnO, Nanostructured Zinc Oxide; c-ZnO, Commercial Zinc Oxide; WPC, Whey Protein Concentrate; WPI, Whey Protein Isolate; FESEM, Field Emission Scanning Electron Microscopy; XRD, X-ray Diffraction; DLS, Dynamic Light Scattering; FTIR, Fourier Transform Infrared Spectroscopy; ATR FTIR, Attenuated Total Reflectance FTIR; MHA, Mueller Hinton Agar; MHB, Mueller Hinton Broth; FFS, Film Forming Suspension; SSA, Specific Surface Area; GRAS, Generally Recognised As Safe
