# Supplementary material for: Whey Proteins–Zinc Oxide Bionanocomposite as Antibacterial Films
Source: Pharmaceutics. 2021 Sep 8;13(9):1426. doi: 10.3390/pharmaceutics13091426 (PMC8466345; doi:10.3390/pharmaceutics13091426)
Supplement: Supplementary file 1 [file pharmaceutics-13-01426-s001.zip › pharmaceutics-1342597-supplementary.pdf]

pharmaceutics

Supplementary Material

Whey Proteins–Zinc Oxide Bionanocomposite as Antibacterial Films

Paolo Pino, Silvia Ronchetti, Chiara Mollea, Marco Sangermano, Barbara Onida* and Francesca Bosco

Department of Applied Science and Technology, Politecnico di Torino, Turin, 10129 , Italy; paolo.pino@polito.it (P.P.); silvia.ronchetti@polito.it (S.R.); chiara.mollea@polito.it (C.M.); marco.sangermano@polito.it (M.S.); francesca.bosco@polito.it (F.B.)

* Correspondence: barbara.onida@polito.it

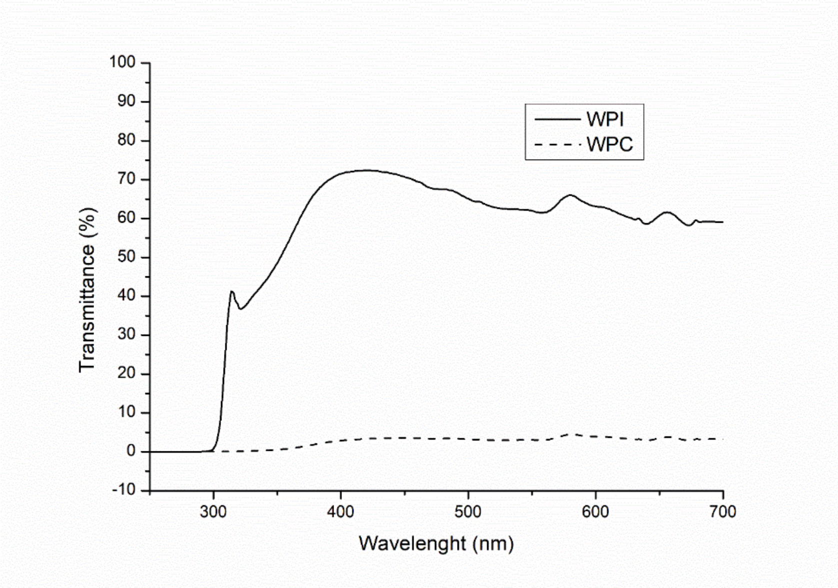

Figure S1. UV-Vis spectra of WPI and WPC films.

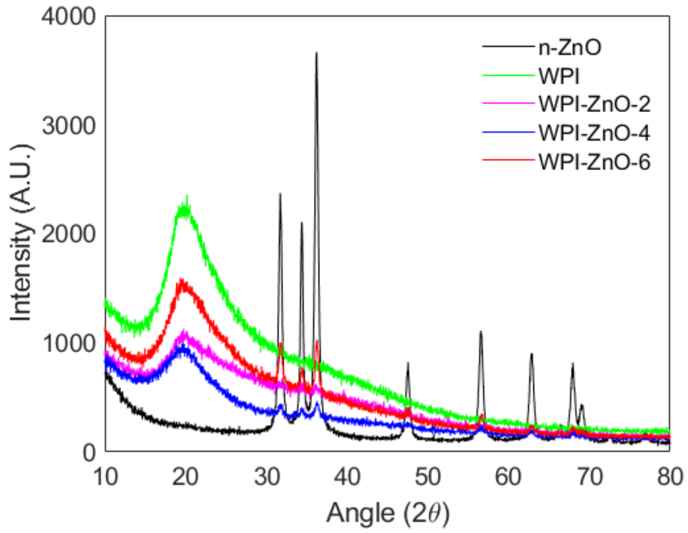

Figure S2. XRD spectra of ZnO, WPI and WPI-ZnO films.
